# The inflammatory cytokine TNF contributes with RAC3-induced malignant transformation

**DOI:** 10.17179/excli2018-1759

**Published:** 2018-11-02

**Authors:** Mileni Soares Machado, Francisco D. Rosa, María C. Lira, Alejandro J. Urtreger, María F. Rubio, Mónica A. Costas

**Affiliations:** 1 Laboratorio de Biología Molecular y Apoptosis, Instituto de Investigaciones Médicas Alfredo Lanari, IDIM-CONICET, Facultad de Medicina, Universidad de Buenos Aires, Combatientes de Malvinas 3150, C1427ARO Buenos Aires, Argentina; 2 Universidad de Buenos Aires, Instituto de Oncología Ángel H. Roffo, Área Investigación, Av. San Martín 5481, C1417DTB Buenos Aires, Argentina; 3 Member of the Consejo Nacional de Investigaciones Científicas y Técnicas (CONICET)

**Keywords:** RAC3, cancer stem cells, mesenchymal cells, TNF-malignant transformation

## Abstract

RAC3 is a coactivator of steroid receptors and NF-κB. It is usually overexpressed in several tumors, contributes to maintain cancer stem cells and also to induce them when is overexpressed in non-tumoral cells. In this work, we investigated whether the inflammatory cytokine TNF may contribute to the transforming effects of RAC3 overexpression in the non-tumoral HEK293 cell line.

The study model included the HEK293 tumoral transformed cell line constitutively overexpressing RAC3 by stable transfection and control non-tumoral cells transfected with an empty vector. The HeLa and T47D tumoral cells that naturally overexpress RAC3 were used as positive control.

We found that TNF potentiated RAC3-induced mesenchymal transition, involving an increased E-Cadherin downregulation, Vimentin and SNAIL upregulation and enhanced migratory behavior. Moreover, concerning the molecular mechanisms by which TNF potentiates the RAC3 transforming action, they involve the IKK activation, which in addition induced the β-Catenin transactivation.

Our results demonstrate that although RAC3 overexpression could be a signal strong enough to induce cancer stem cells, the inflammatory microenvironment may be playing a key role contributing to the migratory and invasive phenotype required for metastasis and cancer persistence.

## Introduction

RAC3 (also known as SRC-3, AIB1, ACTR, p/CIP,TRAM-1), originally identified as a nuclear receptor coactivator and member of the p160 nuclear receptor coactivator's family (Leo and Chen, 2000[18]) is currently considered an oncogene (Ma et al., 2011[23]; Torres-Arzayus et al., 2004[34]). 

Although it was first described as a molecule overexpressed in breast tumors (Yan et al., 2006[41]), it was later discovered as an NF-κB coactivator (Werbajh et al., 2000[38]) overexpressed in a broad spectrum of tumors (Gnanapragasam et al., 2001[12]; Henke et al., 2004[15]; Sakakura et al., 2000[32]) and having additional cytoplasmic functions non-related to its histone acetylase activity (Colo et al., 2008[6]; Ma et al., 2011[23]).

In fact, RAC3 is an oncogene that contributes to tumor development acting as a nuclear receptor coactivator of several transcription factors (Liao et al., 2002[19]; Wang et al., 2013[37]; Yan et al., 2006[41]) that control the expression of genes related to cell cycle and proliferation (Louie et al., 2006[22]; Planas-Silva et al., 2001[28]; Rubio et al., 2006[31]; Torres-Arzayus et al., 2004[34]; Zhou et al., 2003[42]), inhibition of apoptosis (Colo et al., 2007[5], 2008[6])) and autophagy (Fernandez Larrosa et al., 2012[11]). However, additional functions related to tumor progression and metastasis development (Qin et al., 2008[29]; Wang et al., 2013[37]), such as metalloproteinases expression (Qin et al., 2008[29]), cell migration and epithelial-mesenchymal transition (EMT) (Tomar and Schlaepfer, 2010[33]; Wang et al., 2013[37]) have also been described and attributed to the RAC3 splicing variant Delta 4-SRC3 (Long et al., 2010[21]). Interestingly, several of these processes are positively regulated by growth factors (Bedi et al., 2014[3]) and inflammatory cytokines as TNF (Dong et al., 2007[9]; Rubio et al., 2006[31]). In addition, some genes involved in the control of cell cycle, proliferation, apoptosis, cell adhesion and tumor progression are targets of NF-κB, a transcription factor that is activated under inflammatory conditions (Baldwin, 2001[2]; Guttridge et al., 1999[14]; Hinz et al., 1999[16]; Lin and Karin, 2003[20]). Moreover, we have recently demonstrated that inflammatory cytokines upregulate RAC3 expression levels both *in vitro* and *in vivo*, through a direct action of NF-κB on the RAC3 gene promoter (Alvarado et al., 2014[1]).

In addition to its role as an oncogene, it was recently demonstrated that RAC3 expression is required in order to preserve the pluripotency and self-renewal of stem cells (Percharde and Azuara, 2012[26]; Percharde et al., 2012[27]). In normal healthy tissues, the RAC3 expression is downregulated in mature and differentiated cells, suggesting that changes in its expression levels may play a critical role in development. In this regard, the EMT plays a key role not only in tumor progression and metastasis spreading, but also in morphogenesis during embryonic development and tissue repair (Gonzalez and Medici, 2014[13]). In this last case, inflammation usually accompanies the process.

Most of the studies that allowed to define RAC3 as an oncogene were performed in models where it is naturally overexpressed, such as cell lines, tumors and transgenic or knockout mice (Xu and Li, 2003[40]). Although the effect of RAC3 overexpression in non-tumoral cells has not been deeply investigated up to date, we have previously demonstrated that RAC3 overexpression as a unique change, in the non-tumoral human embryonic kidney cell line (HEK293) gives to these cells the ability to grow in soft agar forming colonies (Rubio et al., 2012[30]) and to induce cancer stem cells (CSC) (Panelo et al., 2018[25]).

In this work, we investigated the role of TNF stimulation over the RAC3 overexpression-induced tumoral transformation using an original non-tumoral cell model.

## Materials and Methods

### Cell culture and reagents

The human embryonic kidney HEK293, the human tumoral HeLa and T47D cells were maintained in DMEM (Gibco Laboratories, Grand Island, NY) supplemented with 10 % fetal calf serum (FCS) (Invitrogen), penicillin (100 U/ml) and streptomycin (100 μg/ml). Cells were maintained at 37 °C in a humidified atmosphere with 5 % CO_2_. Unless stated otherwise, all reagents were obtained from Sigma Chemical co. Bs. As., Argentina or Santa Cruz Biotechnology, USA.

### Expression vectors and reporter plasmids

HEK293 cells were transfected with a RAC3 expression vector pCMV-Tag 2B-RAC3 (RAC3) or with empty vector (EV) and selected for stable expression with Neomycin. The tumoral cell lines were transfected with an expression vector for shRNA-RAC3 (pRV-GFP-puromycin) or the scramble control, as previously described in our laboratory and selected for stable expression with puromycin (Panelo et al., 2018[25]).

Reporter plasmids containing the consensus sequence for NF-κB binding (κB-Luc), TCF binding (TOPFlash TCF/β-Cat-Luc) and the IκBss expression vector carrying the mutated IκB at Ser32 and Ser36 to prevent phosphorylation and proteolysis were used as previously described (Fernandez Larrosa et al., 2012[11]; Rubio et al., 2006[31]; Werbajh et al., 2000[38]).

### Immunofluorescence

Immunofluorescence was performed as previously described (Colo et al., 2008[6]). Briefly, HEK293 RAC3 or EV transfectants were seeded on glass coverslips onto 24-well plates in DMEM medium containing 10 % FBS and 24 h later it was replaced by fresh medium and stimulated or not with TNF 20 ng/ml, sulfasalazine 250 μM or TNF plus sulfasalazine (30' before of TNF treatment), during 24 h or as indicated. Then, the cells were fixed with formaldehyde 37 %, permeabilized with PBS-Triton 0.2 %, blocked with 10 % FBS and incubated 2 h at room temperature with 5 μg/ml of anti-β-Catenin antibody (sc:65480) (Santa Cruz Biotechnology). Finally, coverslips were incubated with rhodamine conjugated secondary antibody and visualized with a fluorescence microscope Olympus BX51 and photographed at 1000X magnification.

### Western blot analysis

Western blot assays were performed as previously described (Alvarado et al., 2014[1]). Briefly, total proteins were obtained from HEK293 EV or RAC3 cells stimulated or not for 24 h with four different treatments as described above. Proteins were separated on 8 % SDS-PAGE, and electro-transferred to a nitrocellulose membrane, which was blocked for nonspecific binding with TBS 5 % milk and 0.05 % Tween-20 (T-TBS) and incubated overnight in T-TBS/0.5 % BSA with 0.5 μg/ml of anti-Vimentin, anti-E-Cadherin, or anti-β-Catenin primary antibodies. Subsequently, membranes were washed and incubated for 1 h with a HRP-conjugated secondary antibody, developed by chemiluminescence (Santa Cruz Biotechnology).

### qPCR assay

qPCR assay was performed as previously described (Alvarado et al., 2014[1]). Briefly, total RNA was isolated from HEK293 transfectants treated as described above by using the TRIzol protocol (Invitrogen). Reverse transcription was carried out by using the SuperScript II kit (Invitrogen) following the manufacturer's instructions. For gene expression analysis, qPCR was performed by using sequence-specific primers for:

**RAC3**: **(FW**: aagtgaagagggatctgga, 

**RV**: cagatgactaccatttgagg)

**Vimentin**: (**FW**: gaacctgagggaaactaatctg, **RV**: ctgagaagtttcgttgataacc)

**E-Cadherin**: (**FW**: tggtcaaagagcccttactg, **RV**: caagtcaaagtcctggtcct)

**Snail**: (**FW**: cttccagcagccctacgac, 

**RV**: cggtggggttgaggatct) 

**MMP2**: (**FW**: ccagaataccatcgagacca, 

**RV**: gtagccaatgatcctgtatgtg) and

**GADPH**:** (FW**: tctcctctgacttcaacagc, 

**RV**: gttgtcataccaggaaatga) as an internal control.

### Luciferase assays

The assay was performed as previously described (Alvarado et al., 2014[1]). Briefly, HEK293 EV or RAC3 cells were plated in 24-well plates 24 h prior to transfection at a density of 2.5x10^5^ cells/well. Cells were transiently transfected with a total of 0.2 µg of DNA (0.1 µg of TOPFlash TCF/β-Cat-Luc, 5 ng RSV-β Gal as the control for transfection efficiency and in some experiments, plus IκBss) using the calcium phosphate precipitation method. The medium was replaced after 7 h and cells were stimulated as described above.

The assays for luciferase and β-galactosidase activity were performed after 48 h of treatment using the appropriate substrates following the manufacturer's protocols (Promega Corp. USA). The luciferase values were normalized to the control β-galactosidase (with constitutive expression and activity).

### Wound-healing sssay

The assay was performed as previously described (Del Monaco et al., 2009[8]). Briefly, 1.5x10^6^ cells/cm^2^ HEK293 EV or RAC3 were seeded onto 6-well plates in DMEM medium containing 10 % FBS, until achieving a confluent cell layer. Monolayers were manually scraped with a 200-μl pipette tip and washed with medium without serum to remove non-adherent cells. Then, DMEM without FBS was added to attenuate cellular proliferation without impairing cell survival, following four different treatments: a control group with the drug vehicles, TNF 20 ng/ml, sulfasalazine 250 μM, TNF 20 ng/ml plus sulfasalazine, (30' before of TNF treatment). Cells were cultured at 37 ºC in humid air with constant 5 % CO_2_. Wound scraping was considered time 0 and images of the wounded area were taken under the microscope at 24 h after the injury. Healing was quantified using Image J 1.39 software (Image Processing and Analysis in Java, NIH, Bethesda, MD). Cell migration was expressed as the percentage of the area occupied by the migratory cells in the original cell-free wounded area. The results showed the average ± SD of three different cell assays.

### Metalloproteinases (MMP) activity assay

MMP2 enzymatic activity was determined by zymography, as previously described by Urtreger et al. (2005[35]). Briefly, samples were run on 9 % SDS polyacrylamide slab gels containing 1 mg/ml of gelatin, under non-reducing conditions. After electrophoresis, gels were washed for 30 min using in 2.5 % Triton X-100 and subsequently incubated for 48 h at 37 °C in a buffer containing 0.25M Tris-HCl pH 7.4, 1M NaCl, and 25 mM CaCl_2_. For detection of non-specific activity, the gels were incubated in the same buffer solution but supplemented with 40 mM EDTA. After incubation, gels were fixed and stained with 0.5 % Coomassie Brilliant Blue G-250 in methanol/acetic acid/H2O (30:10:60). The white bands corresponding to MMP2 activity were determined and analyzed using Image J program after Coomassie Blue (Sigma) staining, where the densitometry values for each band was normalized to the protein contents of the conditioned medium as determined by Bradford assay. 

### Statistics analysis 

At least three independent experiments were carried out in all cases. Results were expressed as the mean ± standard deviation (SD). The significance of differences between experimental conditions was determined using ANOVA and the Tukey-Kramer Multiple Comparisons Test for unpaired observations.

## Results

### TNF contributes to RAC3 overexpression-induced mesenchymal transformation of HEK293 cells

We have previously found that RAC3 has a transforming role in HEK293 cells when is overexpressed by stable transfection, acquiring not only the ability to grow in an anchorage-independent manner (Rubio et al., 2012[30]) but also to initiate tumors when are inoculated to mice (Panelo et al., 2018[25]). 

**Figure 1:**
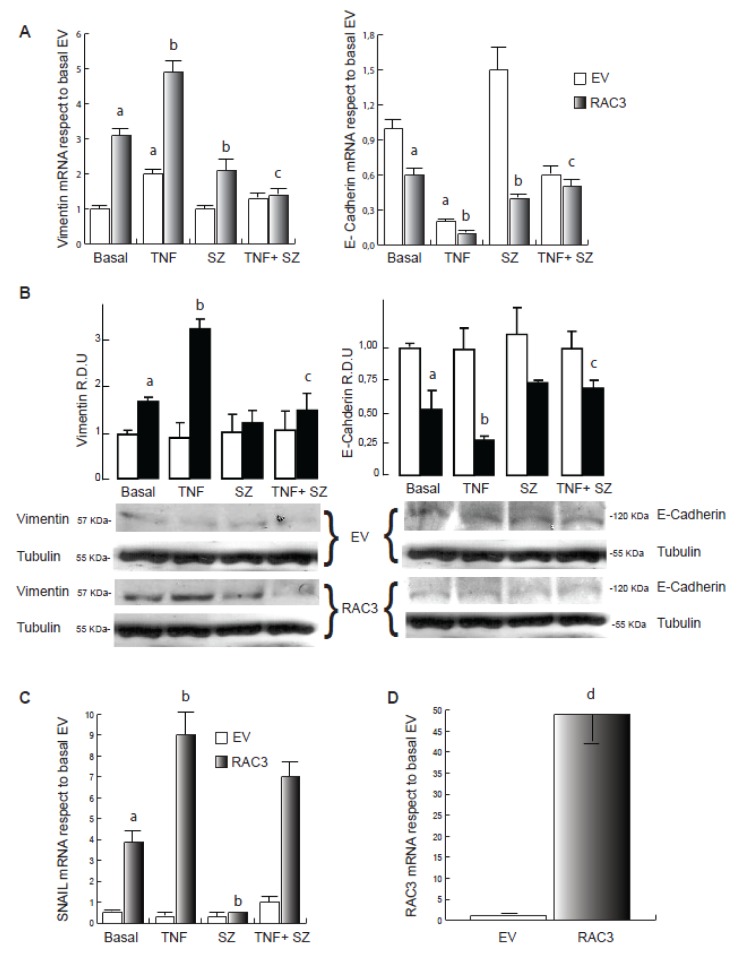
TNF contributes to RAC3 overexpression-induced mesenchymal transformation of HEK293 cells. qPCR analysis for Vimentin and E-Cadherin (A), Snail (C) and RAC3 (D) of total RNA from cells EV (empty vector) and RAC3 (overexpressing RAC3) HEK293 stimulated during 24 h with: vehicle (Basal), TNF 20 ng/ml (TNF), sulfasalazine 250 μM (SZ) and TNF plus sulfasalazine (TNF+SZ). In B, western blot for Vimentin and E-Cadherin showing one representative image and the relative densitometric units (R.D.U) from three independent experiments. a: p < 0.05 respect to basal EV, b: p < 0.05 respect to basal RAC3, c: p < 0.05 respect to TNF stimulated RAC3, d: p < 0.05 respect to cells transfected with empty vector

As previously demonstrated (Panelo et al., 2018[25]), although HEK293 cells express moderated levels of the mesenchymal marker Vimentin, they were significantly increased when RAC3 was overexpressed, while E-Cadherin was significantly inhibited (Figure 1A and B(Fig. 1)). 

TNF is an inflammatory cytokine that activates several transduction signal pathways with multiple biological activities involved in oncogenesis, such as apoptosis, proliferation, angiogenesis, EMT (Colo et al., 2008[6]; Dong et al., 2007[9]; Lee et al., 2007[17]; Rubio et al., 2006[31]) and involves the activation of NF-κB. Interestingly, RAC3 is not only a NF-κB coactivator (Werbajh et al., 2000[38]) but also a target of this transcription factor and is upregulated by TNF (Alvarado et al., 2014[1]). Thus, TNF could be contributing to cell transformation through the increase in RAC3 expression. Therefore, we then investigated the effect of TNF on the transforming role of RAC3 overexpression.

We found that TNF stimulation during 24 h induces the increase of Vimentin levels and the inhibition of E-Cadherin in control cells when analyzed by qRT-PCR. These effects were significantly potentiated by RAC3 overexpression, as determined by qPCR and Western Blot (Figure 1A and B(Fig. 1)). Moreover, the effects on E-Cadherin expression were correlated with changes in the expression pattern of the E-Cadherin inhibitor SNAIL (Figure 1C(Fig. 1)). Figure 1D(Fig. 1) shows that cells transfected with the RAC3 expression vector constitutively overexpress this molecule.

TNF activates IKK, which phosphorylates IκB leading the activation of NF-κB and other substrates such as RAC3 (Wahl et al., 1998[36]; Wu et al., 2002[39]). In order to investigate whether this transduction signal may be involved in the effects of RAC3 and TNF, we performed the same experiments in the presence of sulfasalazine (SZ) a specific IKK inhibitor. We found that SZ addition significantly inhibited the TNF-induced modulation of Vimentin (Fig 1A and B(Fig. 1)) and E-Cadherin (Figure 1A and B right panel(Fig. 1)) having a minor effect on SNAIL in cells overexpressing RAC3 (Figure 1C(Fig. 1)). Interestingly, SZ shows to inhibit the transforming effect of RAC3 overexpression in the absence of TNF stimulation, as detected for mRNA expression of Vimentin, E-Cadherin (Figure 1A(Fig. 1)) and SNAIL (Figure 1C(Fig. 1)). These results demonstrate that TNF potentiates the transforming role of RAC3 overexpression. Concerning the signaling, although both of them involve the NF-κB activation pathway, additional signals could not be excluded.

**Figure 2:**
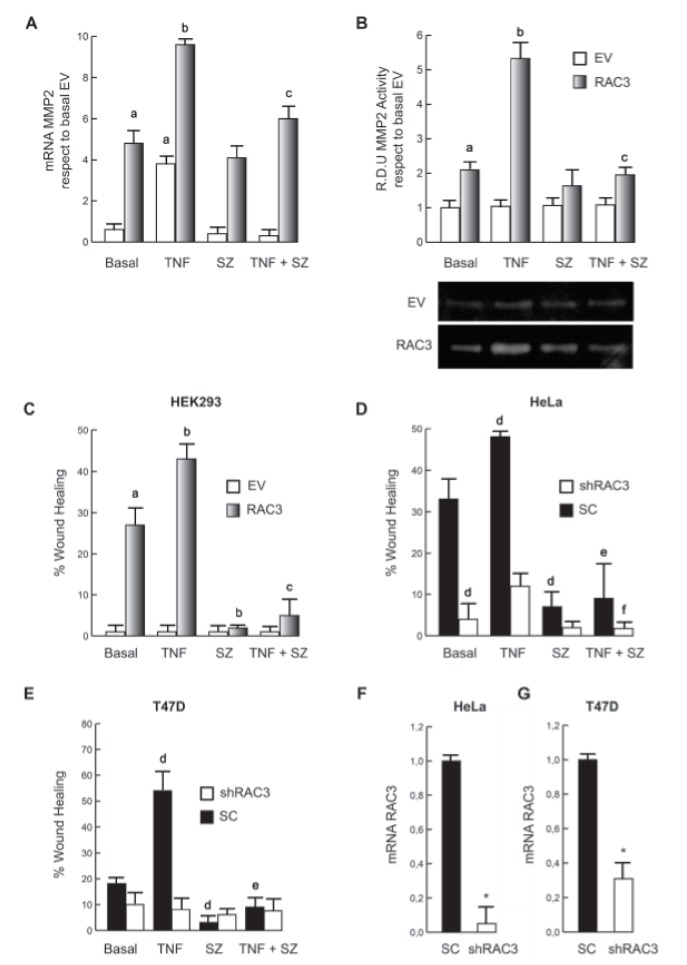
TNF stimulation potentiates RAC3 overexpression-induced cell migration. MMP2 expression was determined by qPCR (A) and activity by zimography assays (B), where the down panel image corresponds to one representative assay from three. The diagram bars show the average +/- SD of wound coverage compared to time 0 and relative to basal EV in HEK293 (C), HeLa (D) and T47D (E). qPCR assays show a significant inhibition of RAC3 expression levels in HeLa (F) and T47D (G) stably expressing shRAC3. a: p < 0.05 respect to basal EV, b: p < 0.05 respect to basal RAC3, c: p < 0.05 respect to TNF stimulated RAC3, d: p < 0.05 respect to basal scramble (SC), e: p < 0.05 respect to TNF stimulated SC, f: p < 0.05 respect to TNF stimulated shRAC3, *p < 0.01 respect to SC.

Then we investigated if the RAC3-induced increase of metalloproteinases production could be also potentiated by TNF. We found that metalloproteinase 2 was significantly increased under simultaneous RAC3 overexpression and TNF stimulation as determined by qPCR (Figure 2A(Fig. 2)) and zymography assays (Figure 2B(Fig. 2)).

As observed for mesenchymal transition, the effect of TNF on metalloproteinases production was significantly affected by IKK inhibition in all the lines. 

### TNF stimulation potentiates RAC3 overexpression induced cell motility

Cancer propagation requires the migration and invasion of CSC and this is promoted by RAC3 overexpression (Panelo et al., 2018[25]). Therefore, as a biological correlation of the transformation induced by RAC3 overexpression and TNF stimulation, we analyzed the migratory potential of both cell clones stimulated or not by this cytokine.

Figure 2C(Fig. 2) shows that although TNF stimulation was unable to induce migration on HEK293 having low normal levels of RAC3, it potentiated the migration induced by RAC3 overexpression.

In order to validate these results in tumoral cells that naturally overexpress RAC3, we analyzed and compared the effect of TNF when RAC3 levels were downregulated by shRAC3 expression in HeLa (Figure 2D(Fig. 2)) and T47D (Figure 2E(Fig. 2)) cells. As shown, the potentiating effect of TNF was similar to that observed in HEK293 cells and diminished when RAC3 was downregulated. Figure 2F-G(Fig. 2) shows that cells transfected with the shRAC3 expression vector, effectively express reduced levels of the coactivator, in agreement with the previously established model using these cells and the same vector (Panelo et al., 2018[25]). Interestingly, the inhibition of NF-κB activity by SZ totally blocked migration.

### TNF stimulation together with RAC3 overexpression induce both ß-catenin nuclear translocation and TCF/ß-Catenin transcriptional activity 

In addition to its structural role in adherent junctions, E-Cadherin mediates the dynamic of β-Catenin, which act as a transcription factor serving as a coactivator of the Tcf/Lef family of DNA-binding proteins (Du and Geller, 2010[10]). In the nucleus, the β-Catenin transcriptional activity involves the expression control of genes related to oncogenesis, proliferation, differentiation, and EMT (MacDonald et al., 2009[24]), interestingly, similar to the biological functions of NF-κB.

In view that RAC3 overexpression downregulates the E-Cadherin and TNF potentiates this effect, we decided to investigate how these signalings affect the β-Catenin and NF-κB transactivation.

**Figure 3:**
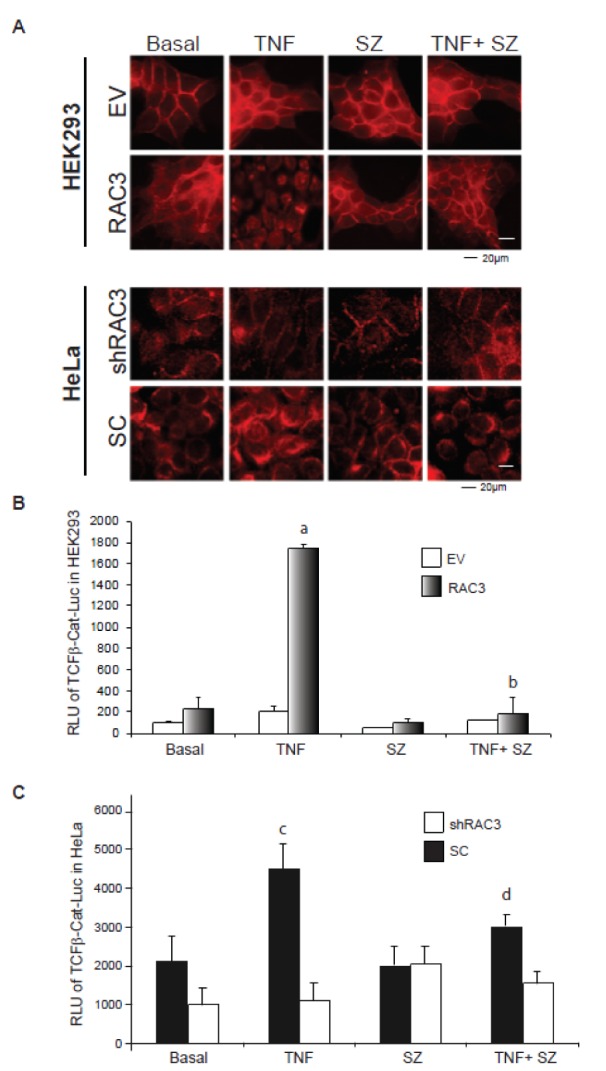
TNF stimulation together with RAC3 overexpression induce the β-catenin nuclear translocation and transcriptional activity of TCF/β-catenin complex A. Inmunofluorescence analysis of β-Catenin in HEK293 (upper panels) and HeLa (bottom panels) stimulated during 24 h with: vehicle (Basal), TNF 20 ng/ml (TNF), sulfasalazine 250 μM (SZ) and TNF plus sulfasalazine (TNF+SZ). B and C-Reporter assays for TCF/β-Catenin-Luc. The bar diagram shows the average relative light units (RLU) of HEK293 (B) and HeLa (C) stimulated with vehicle (Basal), TNF 20 ng/ml, sulfasalazine 250 μM (SZ), TNF plus sulfasalazine (TNF+SZ) and normalized respect to the β-galactosidase activity at each case. a: p < 0.05 respect to basal RAC3, b: p < 0.05 respect to TNF stimulated RAC3, c: p < 0.05 respect to basal SC, d: p < 0.05 respect to TNF stimulated SC.

We investigated the subcellular localization of β-Catenin in cells overexpressing or not RAC3. Our results demonstrate that while in control cells the cytoplasmic pools were detected close to the cell membrane, some nuclear translocation could be detected under RAC3 overexpression condition. Interestingly, under TNF stimulation during 24 h, almost all the β-Catenin was localized in the nuclei of RAC3 overexpressing clones, having only a slight effect in control cells (Figure 3A upper panel(Fig. 3)). The nuclear translocation was inhibited in all the cases by addition of the IKK inhibitor SZ.

Similar results were obtained in HeLa tumoral cells, which naturally overexpress RAC3 (Figure 3A, down panel(Fig. 3)).

Although both NF-κB and β-Catenin pathways are involved in the control of EMT (Dong et al., 2007[9]; Gonzalez and Medici, 2014[13]; MacDonald et al., 2009[24]) previous published works suggest a mutual antagonism (Chang et al., 2013[4]; Du and Geller, 2010[10]; MacDonald et al., 2009[24]). Therefore, we analyzed the TCF/β-Catenin-dependent transcriptional activity using a specific reporter assay.

As shown in Figure 3B(Fig. 3) the TCF/β-Catenin-mediated transcriptional activity was significantly induced only under TNF stimulation and RAC3 overexpressing cells, as observed in the originally non-tumoral HEK293 and the tumoral HeLa cells (Figure 3C(Fig. 3)). In addition, we found that inhibition of NF-κB activation by SZ and transfection with IκBss (data not shown) completely blocked the β-Catenin transactivation in agreement with its effect on β-Catenin nuclear translocation. 

Taken together all these results demonstrate that the transforming signaling induced by TNF under RAC3 overexpression involves at least, the activation of TCF/β-Catenin transcriptional activity, in an IKK-dependent pathway.

## Discussion

Most of the changes in the gene expression pattern involved in EMT have been related to epigenetic regulation (Bedi et al., 2014[3]). In fact, the EMT is required for cell migration and invasion and implies a quick and efficient reversibility when the mesenchymal cells find a new niche where to differentiate and develop a secondary tumoral focus in the case of metastasis, as well as new tissues in embryonal development. 

Certain epigenetic changes in chromatin structure can occur through the exchange of variant histones or assembly and disassembly of chromatin structure via histone chaperones, or through chromatin remodeling proteins. Substantial changes in epigenetic modifications occur to different degrees during various developmental processes such as germ cell development and stem cell differentiation as well as during pathologic processes such as tumorigenesis (Bedi et al., 2014[3]).

RAC3, originally described as a nuclear receptor coactivator and currently considered as an oncogene (Ma et al., 2011[23]; Torres-Arzayus et al., 2004[34]), is a molecule that in addition to some cytoplasmic functions (Colo et al., 2008[6]), has an intrinsic histone acetyl-transferase activity (HAT) and the ability to recruit additional chromatin modifier enzymes like methylases and another HAT factors (Liao et al., 2002[19]). Therefore, when RAC3 is recruited by specific nuclear transcription factors, its principal function in the nucleus is chromatin remodeling and epigenetic control of gene expression.

RAC3 overexpression usually accompanies tumor development which involves several mutations and chromosome aberrations. However, we have previously demonstrated that RAC3 overexpression as a unique genomic change introduced in non-tumoral cells may induce CSC (Panelo et al., 2018[25]). In this work, we found that constitutive RAC3 overexpression affects the cytoskeletal structure, cell motility, and the activation of at least two signal transduction pathways strongly involved in tumorigenesis, metastasis and embryonic development (Baldwin, 2001[2]; Chang et al., 2013[4]; Dong et al., 2007[9]; Gonzalez and Medici, 2014[13]; Lin and Karin, 2003[20]; MacDonald et al., 2009[24]). Thus, we found that RAC3 overexpression promotes the increase of transcriptional activity not only of NF-κB as previously described (Werbajh et al., 2000[38]) but also of β-Catenin mediated transcription. The nuclear translocation and transactivation of β-Catenin can be induced by the Wnt-β-Catenin pathway; however, β-Catenin may also bind E-Cadherin sharing the same domains that are involved in the binding to Tcf/Lef transcription factors. Therefore, when the levels of E-Cadherin are high, β-Catenin is recruited to the transmembrane pool of proteins, inhibiting its nuclear translocation and antagonizing the Wnt-β-Catenin pathway. In addition to its nuclear or transmembrane pool, β-Catenin is also under a constitutive turnover, as part of a cytoplasmic complex where is phosphorylated and degraded (MacDonald et al., 2009[24]).

Although several inflammatory and not inflammatory signals can activate NF-κB, in the case of β-Catenin, the turnover and transcriptional activity are mainly regulated by Wnt activation pathway (MacDonald et al., 2009[24]). 

The tumor microenvironment plays an important role in promoting cancer progression through EMT induction, invasiveness and metastasis and most tumors are infiltrated by immune cells that produce inflammatory cytokines. 

**Figure 4:**
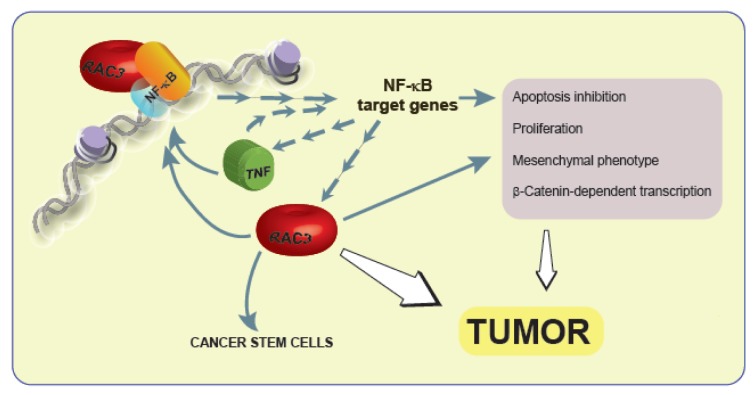
The interplay of RAC3 overexpression and TNF as part of a positive feedback contributes to tumor development The increase of RAC3 that could be induced by inflammatory cytokines is a mesenchymatic transforming signal that increases Vimentin, donwregulates E-Cadherin expression, enhances β-catenin-dependent transcriptional activity and cell migration. These effects are potentiated by TNF and some of them are dependent of NF-κB. However, additional cytoplasmic RAC3 actions or NF-κB independent effects of this coactivator could not be excluded.

TNF is an inflammatory cytokine that activates several transduction signal pathways with multiple biological activities involved in oncogenesis, such as apoptosis, proliferation, angiogenesis and EMT (Colo et al., 2008[6]; Dong et al., 2007[9]; Lee et al., 2007[17]; Rubio et al., 2006[31]) and involves the activation of NF-κB. Interestingly, RAC3 is not only a NF-κB coactivator (Werbajh et al., 2000[38]) but also a target of this transcription factor. Therefore, TNF is able to induce the increase of RAC3 gene expression (Alvarado et al., 2014[1]). Thus, TNF could be contributing to cell transformation through the increase in RAC3 expression, although additional signals could not be excluded (Figure 4(Fig. 4)). Afterward, in this work, we investigated the role of TNF on RAC3 effects, in an experimental model where RAC3 is constitutively overexpressed, in order to become independent of the endogenous RAC3 increase that could be induced by this cytokine. We found that TNF potentiates the transforming effects of RAC3 overexpression, contributing to the mesenchymal phenotype. Moreover, the maximal cell migration, β-Catenin nuclear localization and transcriptional activity were observed in conditions of RAC3 overexpression together with TNF stimulation. These observations are in agreement with previous reports concerning the ability of TNF to activate the Wnt pathway (Coskun et al., 2014[7]) in addition to the positive effect of IKKα on the β-Catenin transcriptional activity (Du and Geller, 2010[10]). In this regard, we observed that most of the transforming effects of RAC3 overexpression alone or together with TNF stimulation were significantly downregulated by addition of the specific IKK inhibitor SZ, including the β-Catenin transcriptional activity. Therefore, although SZ inhibits the activation of several IKK substrates including NF-κB, our results suggest that activation of this transcription factor is a key element for RAC3 plus TNF-induced transformation. However, we found that some transforming effects of RAC3 over-expression without TNF stimulation were also inhibited by SZ, as observed for mesenchymal transformation (Figure 1(Fig. 1)) and cell migration (Figure 2(Fig. 2)). Although these results are in agreement with the previous work demonstrating that RAC3 is an IKK target (Wu et al., 2002[39]), additional signals IKK-independent, downstream of RAC3 could not be excluded.

Taken together all our results, we demonstrate that the increase of RAC3 expression levels affects specific transduction signals by which contributes to the mesenchymal transition and the migratory and invasive behavior of these cells. Interestingly, this transformation could be potentiated by inflammatory cytokines like TNF which share some of the transduction signals affected by RAC3, activating IKK and NF-κB, suggesting that probably this crosstalk could be relevant in primary tumors that overexpress RAC3, usually infiltrated by cells of the immune system that produce inflammatory cytokines *in situ*, favoring the migration and metastasis and increasing the malignancy.

Regarding our present findings, we may conclude that while the overexpression of RAC3 is a mesenchymal/CSC transforming signal, its biological activity is potentiated by TNF, suggesting that inflammatory tumoral microenvironment may be playing a key role in the control of cancer initiation and propagation, contributing to maintain the mesenchymal properties of RAC3-induced CSC.

## Acknowledgements

We thank Dr. Pablo López Bergami (Institute of Biology and Experimental Medicine, Buenos Aires, Argentine), who kindly provided the reporter plasmid TOP/Flash-TCF-β-Cat-Luc.

This work has been supported by grants from the Consejo Nacional de Investigaciones Científicas y Técnicas (CONICET), Instituto Nacional del Cáncer (INC) and Agencia Nacional de Promoción Científica y Tecnológica (ANPCYT). 

## Conflict of interest

The authors have no ethical conflicts to disclose. The authors have no conflicts of interest to declare.
